# 25 years of experience on the management of enterococcal infective endocarditis an observational study

**DOI:** 10.1007/s15010-024-02407-6

**Published:** 2024-10-15

**Authors:** Lorenz Schubert, Rui-Yang Chen, Matthias Weiss-Tessbach, Richard Kriz, Markus Obermüller, Matthias Jackwerth, Wolfgang Barousch, Heinz Burgmann, Manuel Kussmann, Ludwig Traby

**Affiliations:** 1 Department of Medicine I, Clinical Division of Infectious Diseases and Tropical Medicine, Medical University Vienna, Waehringer Guertel 18-20, Vienna, A-1090 Austria; 2 Medical Department with Nephrology & Dialysis, Klinik Ottakring Vienna, Vienna, Austria; 3 Department of Clinical Pharmacology, Medical University Vienna, Vienna, Austria; 4 Department of Laboratory Medicine, Division of Clinical Microbiology, Medical University of Vienna, Vienna, Austria

**Keywords:** *Enterococcus faecalis*, *Enterococcus faecium*, Infective endocarditis, Monotherapy, Mortality

## Abstract

**Purpose:**

As they are effective and well tolerated, aminopenicillins are still the cornerstone for the treatment of enterococcal infections. Current treatment guidelines for infective endocarditis (IE) recommend combination treatments, which carry a higher risk of adverse effects and are based on limited in vitro and experimental data. The aim of this study was therefore to evaluate the treatments of enterococcal IE in real-life practice.

**Methods:**

A total of 4121 episodes of enterococcal bloodstream infections, occurring between 1994 and 2019, were screened for the evidence of IE. Baseline characteristics, risk factors for complicated infections and treatment information were assessed and analyzed using Cox regression analysis.

**Results:**

Overall, 80 (3.9%) IE episodes were identified of which 78 were included in the final analysis. Treatment regimens in our cohort comprised aminopenicillin-monotherapy (*n* = 20), teicoplanin-monotherapy (*n* = 26), other monotherapies (OMT) (*n* = 8), as well as combinations of ampicillin plus daptomycin (*n* = 8), ampicillin plus gentamicin (*n* = 4) or other combinations (*n* = 9). Overall mortality at 28-days was low (9 of 75) and increased to (19 of 75) after 6-months. Frequency of moderate to severe valve regurgitation (*p* = 0.89), or signs of uncontrolled infection (*p* = 0.5) and vegetation size ≥ 10 mm (*p* = 0.11) were similar in the treatment groups. None of the treatment groups was associated with increased hazard for IE-related mortality.

**Conclusions:**

This retrospective study complements previous evidence, demonstrating that monotherapy regimens may be a suitable and effective option for the treatment of IE and supports the need for a prospective evaluation of aminopenicillin-monotherapy for initial and subsequent therapy in these patients.

**Supplementary Information:**

The online version contains supplementary material available at 10.1007/s15010-024-02407-6.

## Introduction

Enterococci are among the leading pathogens of infective endocarditis (IE), accounting for approximately 10% of all cases [1]. Antimicrobial treatment recommendations vary between enterococcal species, namely *Enterococcus faecalis* which is responsible for 90% and *Enterococcus faecium* responsible for 4% of the IE cases [2].

For the treatment of *E. faecalis* IE, current guidelines recommend an antimicrobial combination of two beta-lactam antibiotics (ampicillin plus ceftriaxone) or an aminopenicillin and an aminoglycoside antibiotic for a total treatment duration of four to six weeks [3, 4]. Both antimicrobial combinations have shown synergistic activity in vitro [5, 6]. However, a more detailed examination of the i*n vitro* and in vivo results shows that the synergistic effect is ambiguous and partly dependent on the dosages used in the experiments [5, 7]. Clinical evaluations of aminopenicillin-monotherapy (AMT) compared to combination-therapies (CoT) have been lacking to date [8, 9].

For the treatment of vancomycin susceptible *E. faecium* strains the combination of vancomycin and gentamicin is recommended. In case of vancomycin resistance linezolide, daptomycin, quinupristin-dalfopristin and tigecycline are discussed alternatives [10]. Due to the overall lower rate of *E. faecium* IE, clinical experience is limited.

The aforementioned considerations prompted this retrospective analysis of different antimicrobial regimens for the treatment of enterococcal IE.

## Materials and methods

### Trial design and participants

This retrospective, monocentric observational study was conducted at the University Hospital of Vienna, Austria, a tertiary care hospital. From 1995 to 2019, a total of 4121 positive blood cultures with enterococci were documented in the microbiology database. Positive blood cultures with enterococci occurring within 6 months were defined as one episode, whereas enterococcal BSIs occurring more than 6 months apart were counted as separate episodes.

Thus, 2061 episodes of enterococcal BSI, comprising all 4121 positive blood cultures with enterococci, were included in the analysis and screened twice by different reviewers for the presence of IE. We performed a comprehensive review by hand of all available documentation related to each suspected IE case, including discharge summaries, chart reviews, and records of diagnostic procedures (transthoracic echocardiography, transesophageal echocardiography, PET scans, CT, and MRI), as well as daily physician notes. Potential IE cases were then discussed within the study team, and diagnoses were made according to the modified Duke criteria.

**Fig. 1:**
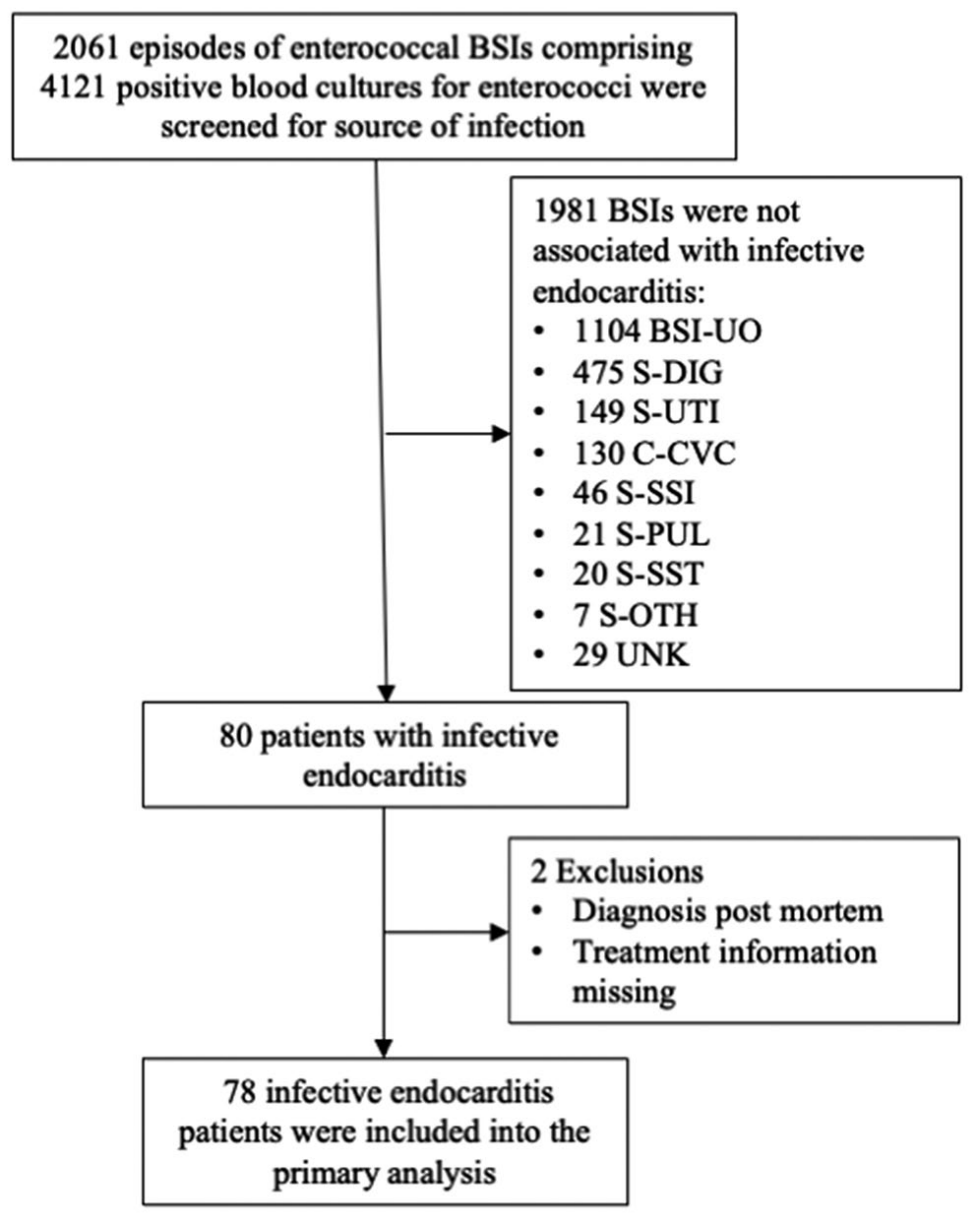
Demonstrates a flow-chart of the work-up of enterococcal bloodstream infections (BSIs). Source of BSI was scored according to European Centre for Disease Prevention and Control (ECDC) [12]. BSI-UO, blood-stream infection unknown origin; S-DIG, blood-stream infection secondary to digestive tract infection; S-UTI, blood-stream infection secondary to urinary tract infection; C-CVC, central-venous catheter infection; S-SSI, blood-stream infection secondary to surgical site infection; S-PUL, blood-stream infection secondary to pneumonia; S-SST, blood-stream infection secondary to skin and soft tissue infection; S-OTH, blood-stream infection secondary to other; UNK, unknown

The other sources of enterococcal BSI episodes were categorized according to the definitions of the European Center for Disease Prevention and Control (ECDC) (Fig. 1) [11, 12].

In addition, the diagnostic workup was evaluated (no BSI workup, general imaging, IE-specific imaging). To assess possible changes in the workup of bloodstream infections over time, arbitrary observation periods were defined and compared based on patient numbers: 1995–2006, 2007–2013 and 2014–2019. The definitions for diagnostic work-up are specified in the supplementary methods.

### Analysis of patients with *Enterococcus* spp. IE

Patients with diagnosed IE were further included in specific analyses. Baseline demographics as well as information on the disease and treatment were obtained from the electronic patient information system (supplementary methods). Patients were then divided into groups based on the anti-enterococcal therapy, namely AMT, teicoplanin-monotherapy, other monotherapies (OMT), ampicillin plus daptomycin, ampicillin plus gentamicin and other treatment combinations. For a correct assessment of mortality (28-day and 6-month mortality), the data were compared with the mortality register of Statistics Austria. Further, autopsy results were screened to determine IE-related mortality, which was defined as death due to any cardiovascular or thromboembolic event. In the absence of autopsy results, the mortality was suspected to be related to IE.

### Statistical analysis

Differences between treatment groups were calculated with the Chi squared test. Numeric data are given as median (quartiles). Univariate and multivariate Cox-regression analyses were performed for predictors of IE-related mortality, namely sex, age, septic embolism, moderate or severe valve regurgitation and vegetation size > 9 mm. Statistical significance was defined as p < 0.05, obtained by two-sided tests. Data collection was supported by Microsoft Excel 365. Statistical analysis as well as graphical presentation was performed in **“**R” version 4.0.3 (R Development Core Team. Vienna, Austria).

## Results

### Screening

**Fig. 2:**
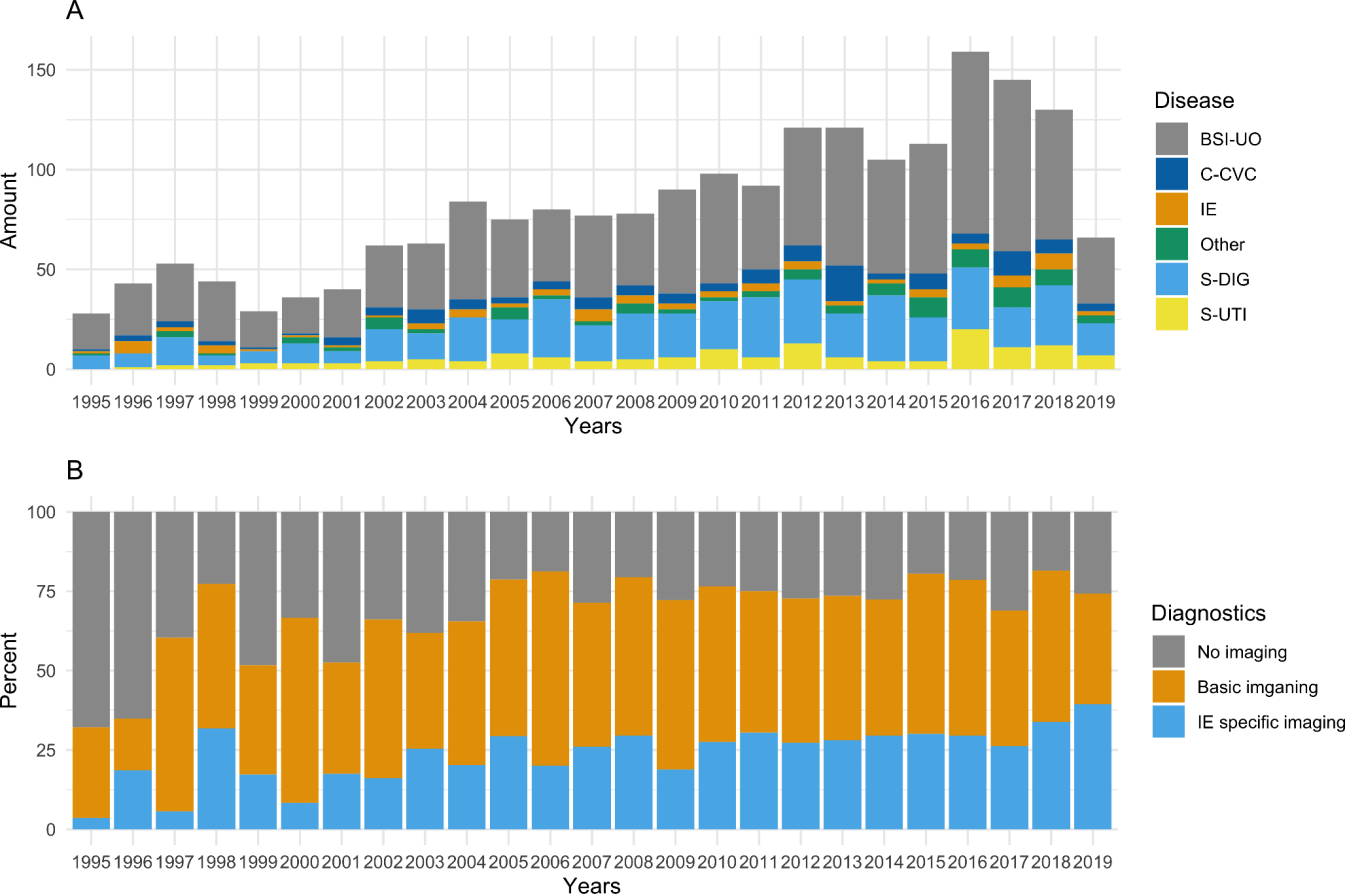
Panel (A) depicts the cause of enterococcal bloodstream infections (BSI). Panel (B) demonstrates the amount of diagnostic work-up performed in order to determine the cause of enterococcal BSI. BSI-UO, blood-stream infection unknown origin; C-CVC, central-venous catheter infection; IE, infective endocarditis; S-DIG, blood-stream infection secondary to digestive tract infection; S-UTI, blood-stream infection secondary to urinary tract infection. Diagnostics are defined as follows: general imaging if at least computed tomography scan, magnetic resonance imaging of abdomen/lungs/brain or ultrasound of abdomen was performed, endocarditis-specific imaging if at least a transthoracic echocardiogram, transesophageal echocardiogram or positron emission tomography was performed and no BSI work-up in none of general imaging or endocarditis-specific imaging was performed

Enterococcal BSIs were screened for the source of infection, and classified into BSIs secondary to digestive tract infections (S-DIG 475, 23.1%), urinary tract infections (S-UTI, 149, 7.2%), central-vascular catheter infections (C-CVC, 130, 6.3%), IE (80, 3.9%), surgical site infections (S-SSI, 46, 2.2%), pulmonary infections (S-PUL, 21, 1%), skin and soft tissue infections (S-SST, 20, 1%) and as BSIs secondary to another source (S-OTH 7, 0.3%). In 1104 episodes of enterococcal BSIs no distinct source of infection was reported (BSI-UO, 53.6%), and in 29 cases patient-specific information could not be obtained (UNK, 1.4%) (Fig. 2, A). There was no significant difference over the periods (1995–2006, 2007–2013, 2014–2019) regarding rates of specific diagnoses (supplementary Table 1).

In 524 (25.8%) patients IE-specific diagnostics were performed, in 1117 (55%) patients at least general imaging was performed and in 391 (19.2%) no BSI work-up was documented (Fig. 2, B). Significantly higher rates of TTE, TEE and relevant radiological imaging were found during assessment periods 2007–2013 and 2014–2019 in comparison to 1995–2006 (supplementary Table 1).

### Analysis of enterococcal IE

Of the 80 episodes of enterococcal IE detected, 78 were included in the final analysis. One enterococcal IE was excluded as the diagnosis was achieved post-mortem and in one patient information on treatment was missing. *E. faecalis* caused 75 (96.2%) and *E. faecium* three (3.8%) of IE cases.

#### *Enterococcus faecalis* related IE

Characteristics of the patients with *E. faecalis* IE are demonstrated in Table 1. Overall mortality at 28-days was 12% (9 of 75) and increased to 25.3% (19 of 75) after 6 months. IE-related mortality at 6 months was 18.7%, with regard to autopsy reports which were available in 50% of included patients (supplementary Table 2).

**Table 1:** Patient characteristics according to the different treatment regimens for *E. faecalis*

		Monotherapy regimens			Combination regimens		
		Ampicillin (*n* = 20)	Teicoplanin (*n* = 26)	Others^A^ (*n* = 8)	Ampicillin plus daptomycin (*n* = 8)	Ampicillin plus gentamicin (*n* = 4)	Others^B^ (*n* = 9)
Age median (Q1– Q3)		67 (62–75)	61 (45–69)	57 (43–72)	75 (69–77)	66 (53–78)	72 (52–77)
Female sex (n[%])		1 (5)	16 (61.5)	6 (75)	3 (37.5)	4 (100)	6 (66.7)
BMI median (Q1– Q3)		26.2 (23–29.2)	24.9 (22.7–26.7)	25.7 (23.3–28.6)	23.4 (21.6–27)	27.9 (26.3–30.1)	24 (23.6–27)
**Comorbidities (n[%])**							
Diabetes mellitus		6 (30)	9 (34.6)	1 (12.5)	2 (25)	1 (25)	3 (33.3)
Cancer		17 (85)	14 (53.8)	3 (37.5)	8 (100)	3 (75)	6 (66.7)
Cardiac disease		5 (25)	3 (11.5)	0	2 (25)	0	2 (22.2)
IV drug abuse		2 (10)	1 (3.8)	2 (25)	0	0	2 (22.2)
**Cardiac involvement (n[%])**							
Mitral valve		4 (20)	6 (23.1)	3 (37.5)	3 (37.5)	1 (25)	2 (22.2)
Aortic valve		9 (45)	14 (53.8)	3 (37.5)	3 (37.5)	2 (50)	4 (44.4)
Mitral and aortic valve		3 (15)	3 (11.5)	1 (12.5)	1 (12.5)	1 (25)	2 (22.2)
Other location		2 (10)	2 (7.7)	1 (12.5)	0	0	1 (11.1)
Prosthetic valve IE		8 (40)	5 (19.2)	4 (50)	3 (37.5)	1 (25)	3 (33.3)
Device related IE		0	1 (3.8)	0	1 (12.5)	0	0
Vegetation size > 9 mm		5 (25)	14 (53.8)	3 (37.5)	2 (25)	3 (75)	3 (33.3)
Septic embolism		9 (45)	12 (46.2)	5 (62.5)	2 (25)	1 (25)	3 (33.3)
Moderate/severe valve regurgitation		11 (55)	12 (46.2)	5 (62.5)	4 (50)	1 (25)	5 (55.6)
**Surgical treatment**							
Surgical treatment received– yes(n[%]		7 (35)	11 (42.3)	3 (37.5)	1 (12.5)	1 (25)	5 (55.6)
Time to surgical intervention in days median (Q1– Q3)		26 (13–49)	14 (3–41)	23 (8–64)	6	33	12 (0–15)
**Fulfilled duration of initial treatment n(%)** ^C^							
7 days		20 (100)	22 (84.6)	8 (100)	8 (100)	4 (100)	8 (88.9)
14 days		17 (85)	21 (80.8)	8 (100)	6 (75)	3 (75)	6 (66.7)
21 days		15 (75)	18 (69.2)	6 (75)	5 (62.5)	3 (75)	5 (55.6)
28 days		12 (60)	17 (65.4)	4 (50)	4 (50)	3 (75)	4 (44.4)
6 weeks		6 (30)	13 (50)	3 (37.5)	3 (37.5)	1 (25)	1 (22.2)
No information on treatment duration		0	4 (15.4)^D^	0	0	0	0
**Completion treatment regimens n(%)** ^´E^							
Adap Ad Adaptation of treatment		11 (55)	5 (19.2)	2 (25)	3 (37.5)	2 (50)	6 (66.7)
Amoxicillin/Ampicillin		3 (27.3)	2 (40)	0	1 (33.3)	0	3 (50)
Teicoplanin or Dalbavancin		7 (63.6)	1 (20)	1 (50)	0	1 (50)	2 (33.3)
Linezolid, orally administered		0	0	1 (50)	0	1 (50)	0
Others^F^		1	2 (40)	0	2 (66.7)	0	1 (16.7)
Overall antimicrobial treatment duration days median (Q1– Q3)		45 (33–60)	52 (34–69)	46 (24–59)	62 (37–79)	44 (38–50)	43 (31–48)
**Microbiology**							
Control blood cultures performed yes (n[%])		16 (80)	23 (88.5)	7 (87.5)	8 (100)	4 (100)	7 (77.8)
Control interval days (median[Q1– Q3])		25.5 (5–96)	53 (13–104)	48 (13–201.5)	68 (14.5–155)	64 (42.5–144)	23.5 (8.5–164.5)
Number of blood cultures– median (Q1– Q3)		3 (1.5–4)	4.5 (2–7)	2.5 (1.5–7)	6 (3.5–8)	6.5 (3.5–11)	1 (1–8)
Documented blood culture clearance n(%)		14 (87.5)	20 (87)	5 (71.4)	6 (75)	4 (100)	6 (85.7)
Blood culture recurrence n(%)		1 (7)	2 (10)	1 (20)	3 (50)	0	1 (16.7)
**Mortality n(%)**							
Overall 28-day mortality		2 (10)	3 (11.5)	1 (12.5)	1 (12.5)	0	2 (22.2)
Overall 6-month mortality		6 (30)	6 (23.1)	1 (12.5)	2 (25)	1 (25)	3 (33.3)
IE-associated mortality at 6 months		3 (15)	6 (23.1)	0	2 (25)	1 (25)	2 (22.2)

Q1-Q3, Quartile 1 to Quartile 3; BMI, body mass index; IV drug abuse, intravenous drug abuse; IE, infective endocarditis

^A^ Other monotherapy regimens comprise daptomycin (*n* = 3), linezolid (*n* = 3) and dalbavancin (*n* = 2)

^B^ Other combination regimens comprise ampicillin plus ceftriaxone (*n* = 3), ampicillin plus vancomycin/teicoplanin (*n* = 3), ceftriaxone plus daptomycin (*n* = 1), and teicoplanin plus netilmicin (*n* = 2)

^C^ Initial treatment was defined as treatment which was initiated after diagnosis of infective endocarditis

^D^ Treatment was continued from physicians but no end of treatment documented

^E^ Completion treatment was defined as treatment initiated after the initial treatment

^F^ Other completion treatment regimens comprise daptomycin (*n* = 2), ampicillin plus daptomycin (*n* = 1), amoxicillin plus dalbavancin (*n* = 1), ampicillin plus cefotaxime (*n* = 1) and piperacillin/tazobactam plus netilmicin (*n* = 1)

Risk factors for IE-related mortality were compared between AMT, OMT and CoT. In the univariate analysis, age > 65a was significantly associated with an increased hazard ratio (HR) for IE-related mortality (HR 1.1, 95% CI (1–1.11), p-value = 0.043). Further, there was a trend towards an increased HR for vegetation size > 9 mm (HR 1.2, 95% CI (0.99–1.4), p-value = 0.058), and a trend towards a decreased HR for male sex (HR 0.37, 9% CI (0.13–1), p-value = 0.06) (supplementary Table 3). The association was not confirmed in the multivariate analysis.

After 28 days, 2 patients in the AMT group (10%), 4 in the OMT group (11.8%) and 3 in the CoT group (14.3%) had died. After 6 months, the number of deaths increased to 6 in the AMT group (30%), 7 in the OMT group (20.6%) and 6 in the CoT group (28.6%), of which 3 were IE-associated in the AMT group (15%), 6 in the OMT group (17.7%) and 5 in the CoT group (23.8%). In the univariate analysis, none of the therapies was associated with an increased hazard for IE-related mortality (supplementary Table 4). A Kaplan Meier plot comparing treatment regimens is demonstrated in the supplementary Fig. 1. Antibiotic dosages in dependence of kidney function are displayed in supplementary Table 5 in mg per kg bodyweight. Teicoplanin was administered with a loading dose and guided by therapeutic drug monitoring. Switch to oral treatment was performed after a median of 41.27 days (Q1-Q3 15–43).

Further, control blood cultures were performed in 16 patients in the AMT group (80%), 30 in the OMT group (88.2%), and 19 in the CoT group (90.5%). Of those, rates of recurrent positive blood culture were reported in one patient in the AMT group (7%), 3 patients in the OMT group (10%) and 4 patients in the CoT group (21.1%).

#### Enterococcus faecium related IE

The age of patients with *E. faecium* IE was 52, 61 and 75 years and two (66.7%) were women. Coexisting cardiac disease was reported in two patients (66.7%) and cancer in one patient (33.3%). A vegetation size of > 9 mm was reported in 2 of the patients (66.7%) and 1 patient (33.3%) suffered from moderate to severe valve regurgitation. Signs of uncontrolled infection were present in 2 patients (66.7%). None of the patients received surgical intervention. Antimicrobial regimes were teicoplanin (*n* = 1), daptomycin (*n* = 1) and the combination of daptomycin, gentamicin and ceftazidime/avibactam (*n* = 1). The patient receiving teicoplanin died due to IE-related heart failure.

## Discussion

Enterococcal BSIs have the highest prevalence of IE among gram-positive pathogens typically associated with IE [1]. In addition, the burden of disease caused by enterococcal infections is considerable, with mortality rates of up to 20%, especially in patients with comorbidities or in the case of antibiotic-resistant isolates [3, 4]. In agreement, we report an overall mortality in this cohort of 12% at day 28, and 25.3% at 6 months (18.7% IE-related mortality at 6 months) [3, 4].

In our study, aminopenicillins were administered as initial monotherapy in 20 patients, representing, to our knowledge, the largest cohort of patients treated with AMT reported to date. We did not find an increased IE-associated mortality or risk of recurrent infection in patients receiving AMT in comparison to OMT or CoT. These findings are in contrast with those of Danneels et al., who reported a higher rate of IE relapse after 6 months (33.3%) and higher 6-month mortality (33.3%) in the amoxicillin group. However, their study included only nine patients on amoxicillin monotherapy, of whom six completed the treatment [8].

Despite the limited evidence comparing high-dose AMT to beta-lactam/beta-lactam or beta-lactam/aminoglycoside combination therapies, current guidelines advocate for the use of treatment combinations. These recommendations are based on presumed in vitro and in vivo synergistic effects [9].

In vitro synergism of penicillin plus streptomycin was reported by Jawetz et al. in nine enterococcal strains using time kill curves. Streptomycin alone had no demonstrable activity on enterococci. However, the bactericidal activity of penicillin plus streptomycin was even higher than optimized concentrations of penicillin alone [6]. In vitro synergism of 3rd generation cephalosporins with amoxicillin was only demonstrated at non-bactericidal concentrations of amoxicillin and decreased to comparable but not superior results at higher concentrations of amoxicillin [5, 7]. Transferred into an experimental aortic valve endocarditis model in rabbits, the synergistic effect of 3rd generation cephalosporins in combination with amoxicillin was not shown [5]. Using the same model in rabbits, the synergistic effect of ampicillin plus gentamicin demonstrated a higher rate of sterilization of vegetations on day 7 (ampicillin 5% versus ampicillin plus gentamicin 32%) but had no significant effect on survival (ampicillin 91% versus ampicillin plus gentamicin 92%) or blood culture clearance over time [7]. One explanation for difficulties in transferability of in vitro data may be explained by biofilm formation. Using confocal laser scanning microscopy in an in vitro model, Thieme et al. were unable to demonstrate visible biofilm eradication by ampicillin alone or combination therapies (ampicillin plus ceftriaxone or ampicillin plus gentamicin) in five enterococcal isolates from IE patients [13].

The aforementioned discrepancies of in vitro and in vivo studies, warrant a critical reevaluation of combination therapy for treatment of *E. faecalis* IE. Current guidelines favor beta-lactam/beta-lactam over beta-lactam/aminoglycoside combinations, due to lower risk of renal failure and necessity of treatment discontinuation. Nevertheless, use of cephalosporins may pose an excess risk of *Clostridioides difficile* associated diarrhea and induction of ESBL producing Gram-negative bacteria [14]. In addition, Thieme et al. showed that higher ceftriaxone concentrations (two times the MIC) compared with ampicillin alone favored the selection of enterococcal small colony variants (SCVs). Enterococcal SCVs have been increasingly described in IE cases, indicating higher rates of antimicrobial resistance, particularly to aminoglycosides, compared with the normal phenotype [13].

Teicoplanin, the most frequently initiated monotherapy in this cohort, demonstrated better results as first-line therapy than previously described in terms of overall mortality (23.1% versus 44%, respectively) [15]. In one Spanish trial the authors used teicoplanin, at a median dose of 10 (Q1-Q3, 10–10.8) mg/kg/day following an induction dose of twice-daily teicoplanin, as first-line or salvage therapy for the treatment of IE due to *E. faecalis* (overall [64%], first-line therapy group [11%]) and *E. faecium* (overall [36%], first line therapy group [89%]). The authors argued that high rates of mortality in the first-line therapy arm occurred in patients with an indication for surgery that was not performed. Lower rates of overall mortality in our cohort in comparison to the cohort of Escolà-Vergé et al. may be explained by a higher frequency of surgical interventions (42.3% versus 11%), different enterococcal species (100% *E. faecalis* versus 89% *E. faecium*) and possibly higher teicoplanin dosing. However, teicoplanin serum levels were not described in the Spanish study, whereby no comparison was possible [15].

In this cohort recurrent infections were unevenly distributed and partially exceeded the expected rates of 5 to 7%, with remarkably high rates in the ampicillin plus daptomycin group [3, 4]. Clinical success rates of daptomycin treatment of enterococcal infections were found to be dependent on daptomycin dosage with 85.7% success rate at doses > 8 mg/kg/day compared to 75.8% at doses < 8 mg/kg/day [16]. The dosage recommendations are also supported by in vitro studies. Daptomycin concentrations of > 10 mg/kg demonstrated the most significant and sustained bactericidal activity [17]. Treatment with lower daptomycin dosages (6-8 mg/kg/day) might be influenced by the inoculum effect and therefore less effective [18]. In the present study, the median daptomycin dosage was 10.6 mg/kg/day [Q1-Q3 = 8.9–11.2]) which is in line with suggested high-dosage regimes. The remarkably high rates of recurrent BSIs in the ampicillin plus daptomycin group may be explained by the high rates of prosthetic valve or device-related IE (12.5%) and prosthetic valve IE (37.5%) in this group, while only one patient received surgical intervention. Of note, despite higher numbers of recurrent infections, we did not find high rates of IE-related mortality in this cohort.

At our center, switch to an oral antimicrobial treatment was performed after a median of 41.3 days (Q1– Q3, 15–43). This is later than previously described by Iversen et al., who demonstrated that switch to an antimicrobial agent can be performed after 17 days (Q1-Q3, 14–25) [19].

The specific strengths and limitations of this study need to be addressed. In this analysis, 4% of the enterococcal BSIs were diagnosed as IE. This is significantly less than the rate of *E. faecalis* IE among BSIs reported by Ostergaard et al. in Denmark (16.1%) and Billington et al. in Canada (17%) [1, 20]. Even higher rates (26%) were reported by Dahl et al. who prospectively screened 344 patients with *E. faecalis* BSI by echocardiography for signs of infective endocarditis [21]. Only a quarter of our patients had IE-specific imaging, which may account for the difference. The prevalence of IE in patients receiving IE-specific imaging was 15%, which is comparable to the rates reported in the retrospective studies. Assessing the quality of diagnosis retrospectively over a long period is difficult as diagnostics such as bed-side echocardiography, a common tool on internistic wards, might have been missed if not clearly documented. This applies in particular to patients treated before the implementation of the electronic patient documentation system. At that point most of the patient history, therapy and imaging were handwritten and scanned manually into the medical history. To address this issue the whole cohort was screened twice to minimize the risk of overlooking findings. Of note, we did not detect an increase in diagnosed rates in more recent years. Hence, we believe that the long observational period is a major strength in this study as it allowed a precise and comprehensive description of the enterococcal BSI work-up and used treatment regimens for enterococcal IE. Another surprising finding is that aside from age, none of the risk factors for IE was associated with increased risk for IE-related mortality. We believe this is attributed by the relatively small cohort as well as low rate of IE-related mortality.

## Conclusion

Considering the therapeutic equivalence observed in this study comparing AMT to OMT and CoT, the use of antimicrobial monotherapies for the treatment of enterococcal IE could reduce the occurrence of adverse drug reactions and also facilitate outpatient continuation of treatment. Prospective clinical trials investigating the efficacy of aminopenicillin monotherapies compared to established combination therapies are therefore warranted.

## Electronic supplementary material

Below is the link to the electronic supplementary material.

Supplementary Material 1

## Data Availability

The datasets analyzed for this study are available from the corresponding author on reasonable request.
