# Supplementary material for: 25 years of experience on the management of enterococcal infective endocarditis an observational study
Source: Infection. 2024 Oct 15;53(1):467–74. doi: 10.1007/s15010-024-02407-6 (PMC11825611; doi:10.1007/s15010-024-02407-6)
Supplement: Supplementary file 1 — Supplementary Material 1 [file 15010_2024_2407_MOESM1_ESM.docx]

**Supplementary information**

**Supplements to:**

**25 years of experience on the management of enterococcal infective endocarditis an observational study**

**Supplementary to Material and Methods**

**Assessment of the quality of diagnostic work-up:**

To assess the quality of diagnostic work-up of *Enterococcus spp.* bloodstream infections the diagnostic effort involved was surveyed. The groups were defined as follows:

1. General imaging performed: computed tomography scan or magnetic resonance imaging of abdomen/lungs/brain, ultrasound of the abdomen
2. Infective endocarditis specific imaging performed: transthoracic echocardiogram, transesophageal echocardiogram and positron emission tomography
3. No BSI work-up: absence of general imaging or endocarditis specific imaging

**Baseline parameters extracted from the electronic patient information system for patients with infective endocarditis:**

1. Comorbidities
2. Echocardiography findings: valves involved, size of vegetation, quantification of valve regurgitations
3. Imaging findings for evidence of uncontrolled infection: embolic complications, extracardiac abscesses, or evidence of locally uncontrolled infection such as evidence for pseudoaneurysms and/or fistulae
4. Microbiological findings: blood culture results, frequency of blood cultures performed, blood culture clearance, recurrent infection (BSI occurred >48h after the last negative blood culture regardless the treatment status)
5. History of antimicrobial treatment: agents administered, duration of therapy, required therapy adjustment
6. Surgical procedures performed

**Supplementary to Results**

**Supplementary tables**

|  | | **1995 – 2006**  **(n=637)** | **2007 – 2013**  **(n=677)** | **2014 – 2019**  **(n=718)** | **p-value** |
| --- | --- | --- | --- | --- | --- |
| **Source of infection** | |  |  |  |  |
|  | **IE** | 28 (4.4) | 26 (3.8) | 24 (3.3) | 0.6 |
|  | **BSI-UO** | 352 (55.3) | 354 (52.3) | 398 (55.4) | 0.43 |
|  | **S-DIG** | 152 (23.9) | 171 (25.3) | 152 (21.2) | 0.19 |
|  | **S-UTI** | 41 (6.4) | 50 (7.4) | 58 (8.1) | 0.51 |
|  | **C-CVC** | 38 (6) | 53 (7.8) | 39 (5.4) | 0.16 |
|  | **Other** | 26 (4.1) | 23 (3.4) | 47 (6.5) | 0.014 |
| **IE diagnostic performed** | |  |  |  |  |
|  | **TTE** | 113 (17.7) | 157 (23.2) | 191 (26.6) | <0.001 |
|  | **TEE** | 35 (5.5) | 57 (8.4) | 52 (7.2) | 0.12 |
|  | **PET-CT** | 3 (0.5) | 24 (3.5) | 33 (4.6) | <0.001 |
|  | **Relevant radiological imaging^A^** | 377 (59.2) | 469 (69.3) | 514 (71.6) | <0.001 |
| **No diagnostic performed** | | 163 (25.6) | 114 (16.8) | 115 (16) | <0.001 |

**Supplementary table 1** demonstrates source of *Enterococcus spp.* blood stream infections (BSIs) and the BSI workup throughout the years. IE, infective endocarditis; BSI-UO, bloodstream infection of unknown origin; S-DIG, bloodstream infection secondary to digestive tract infection; S-UTI, bloodstream infection secondary to urinary tract infection; C-CVC, catheter-related infection related to central vascular catheter; Other, not categorized as above; TTE, transthoracic echocardiogram; TEE, transesophageal echocardiogram; PET, positron emission tomography; CT, computed tomography; MRI, magnetic resonance imaging.

^A^ Relevant radiological imaging is defined as imaging that provides support for IE diagnosis by assessing signs of uncontrolled infection (CT scan or MRI of abdomen/lungs/brain, ultrasound of abdomen)

|  | | **Monotherapy regimens** | | | | | | **Combination regimens** | | | | | |
| --- | --- | --- | --- | --- | --- | --- | --- | --- | --- | --- | --- | --- | --- |
|  | | Ampicillin  (n=6) | | Teicoplanin  (n=7) | | Others^B^  (n=1) | | Ampicillin plus daptomycin  (n=2) | | Ampicillin plus gentamicin  (n=1) | | Others^C^  (n=3) | |
|  | | Autopsy performed | | Autopsy performed | | Autopsy performed | | Autopsy performed | | Autopsy performed | | Autopsy performed | |
| Cause of death | | yes | no | yes | no | yes | no | yes | no | yes | no | yes | no |
| IE related mortality | | 1 (16.7) | 2 (33.3) | 4 (57.1) | 3 (42.9) | 0 | 0 | 1 (50) | 1 (50) | 1 (100) | 0 | 1 (33.3) | 1 (33.3) |
|  | IE related heart failure | 1 (16.7) | 1 (16.7) | 2 (28.6) | 2 (28.6) | 0 | 0 | 1 (50) | 1 (50) | 0 | 0 | 1 (33.3) | 1 (33.3) |
|  | Myocardial  infarction | 0 | 0 | 0 | 1 (14.3) | 0 | 0 | 0 | 0 | 1 (100) | 0 | 0 | 0 |
|  | Multiorgan  disorder | 0 | 1 (16.7) | 1 (14.3) | 0 | 0 | 0 | 0 | 0 | 0 | 0 | 0 | 0 |
|  | Uncontrolled  infection | 0 | 0 | 1 (14.3) | 0 | 0 | 0 | 0 | 0 | 0 | 0 | 0 | 0 |
| Non-IE related mortality | | 1 (16.7) | 2 (33.3) | 0 | 0 | 0 | 1 (50) | 0 | 0 | 0 | 0 | 1 (33.3) | 0 |
|  | Bleeding | 0 | 0 | 0 | 0 | 0 | 1 (50) | 0 | 0 | 0 | 0 | 1 (33.3) | 0 |
|  | Perforation of  coecum | 1 (16.7) | 0 | 0 | 0 | 0 | 0 | 0 | 0 | 0 | 0 | 0 | 0 |
|  | Died after  discharge | 0 | 2 (33.3) | 0 | 0 | 0 | 0 | 0 | 0 | 0 | 0 | 0 | 0 |

**Supplementary table 2** demonstrates cause of death in dependence on treatment regimens. Infective endocarditis (IE) related mortality was defined as death due to any cardiovascular or thromboembolic event.

| Risk factors | AMT (n=20) | OMT(n=34) | CoT (n=21) | p-value |
| --- | --- | --- | --- | --- |
| Moderate to severe valve regurgitation | 11 (55) | 17 (50) | 10 (47.6) | 0.89 |
| Signs for uncontrolled infection | 11 (55) | 17 (50) | 6 (28.6) | 0.5 |
| vegetation size >9mm | 5 (25) | 17 (50) | 8 (38.1) | 0.11 |
| Surgical intervention | 7 (35) | 14 (41.2) | 7 (33.3) | 0.82 |

**Supplementary table 3** demonstrates distribution of risk factors and the results from the Chi-squared test in between the treatment groups. AMT, aminopenicillin-monotherapy, OMT, other monotherapies; CoT, combination-therapies.

|  | | HR (95% CI) | p-value |
| --- | --- | --- | --- |
| Risk factors | | | |
|  | >65a | 1.1 (1 – 1.11) | 0.043 |
|  | male sex | 0.37 (0.13 – 1) | 0.06 |
|  | vegetation size >9mm | 1.2 (0.99 – 1.4) | 0.058 |
|  | severe valve regurgitation | 0.96 (0.34 – 2.7) | 0.94 |
|  | uncontrolled infection | 1.3 (0.45 – 3.7) | 0.64 |
| Treatment | | | |
|  | AMT | 0.71 (0.2 – 2.5) | 0.6 |
|  | OMT | 0.91 (0.32 – 2.6) | 0.86 |
|  | CoT | 1.5 (0.5 – 4.5) | 0.47 |

**Supplementary table 4** demonstrates hazard ratios of the univariate analysis for the risk factors and IE associated mortality. AMT, aminopenicillin-monotherapy, OMT, other monotherapies; CoT, combination-therapies.

| Dosage | Ampicillin (mg/kg per Day) | Amoxicillin (mg/kg per Day) | Ceftriaxone (mg/kg per Day) | Teicoplanin (mg/kg per Day) | Vancomycin (mg/kg per Day) | Dalbavancin (mg/kg per Day) | Linezolid (mg/kg per Day) | Daptomycin (mg/kg per Day) | Gentamicin (mg/kg per Day) |
| --- | --- | --- | --- | --- | --- | --- | --- | --- | --- |
| Normal kidney function | 125.7  (105.8 – 159) | 98.8  (81.6 – 100) | 41.2  (26 – 54.8) | 14.7  (13 – 17.7) | 23.5  (23 – 26.7) | 12.9  (4 – 21.7) | 15.8  (11.9 – 23.1) | 10.6  (8.9 – 11.2) | 3.4  (2.5 – 3.6) |
| GFR 30 – 50 ml/min | 143.9  (106.5 – 155.8) | 142.2  (115.4 – 169) | 54.1 | **n.a.** | 21.1 | **n.a.** | **n.a.** | 9.6 | **n.a.** |
| GFR 10 – 29 ml/min | 55  (48.8 – 61.2) | **n.a.** | **n.a.** | 15.8  (11.3 – 20.3) | **n.a.** | **n.a.** | 15.6 | 6.9 | **n.a.** |
| GFR <10 ml/min | **n.a.** | **n.a.** | **n.a.** | **n.a.** | **n.a.** | **n.a.** | **n.a.** | **n.a.** | **n.a.** |
| Dialysis | 36.4 | **n.a.** | **n.a.** | 14.5 | 12.4 | **n.a.** | **n.a.** | 12.7 | **n.a.** |
| Drug monitoring | **n.a.** | **n.a.** | **n.a.** | 20.2  (18 – 25) | 13.1  (10.5 – 15.7) | **n.a.** | **n.a.** | **n.a.** | **n.a.** |

**Supplementary table 5** shows the dosage of antibiotic treatment in mg per kg body weight per day and based on the glomerular filtration rate (GFR) according to the Chronic Kidney Disease Epidemiology Collaboration (CKD-EPI)[25]. N.a., not applicable.


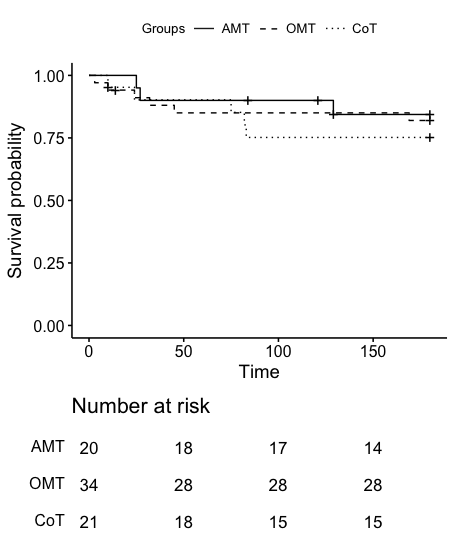


**Supplementary figure 1**, demonstrates the Kaplan Meier plot for infective endocarditis related mortality according to the different treatment regimes, namely aminopenicillin monotherapy (AMT) (n=20), other monotherapies (OMT) (teicoplanin [n=26], daptomycin [n=3], linezolid [n=3] and dalbavancin [n=2]) and combination-therapies (CoT) (ampicillin plus daptomycin [n=8], ampicillin plus gentamicin [n=4], ampicillin plus ceftriaxone [n=3], ampicillin plus vancomycin/teicoplanin [n=3], teicoplanin plus netilmicin [n=2] and ceftriaxone plus daptomycin [n=1].
